# Comparative Genomics Reveals a Remarkable Biosynthetic Potential of the *Streptomyces* Phylogenetic Lineage Associated with Rugose-Ornamented Spores

**DOI:** 10.1128/mSystems.00489-21

**Published:** 2021-08-24

**Authors:** Yoon-Hee Chung, Hiyoung Kim, Chang-Hun Ji, Hyun-Woo Je, Dongho Lee, Sang Hee Shim, Hwang-Soo Joo, Hahk-Soo Kang

**Affiliations:** a Department of Biomedical Science and Engineering, Konkuk University, Seoul, South Korea; b Department of Plant Biotechnology, College of Life Sciences and Biotechnology, Korea University, Seoul, South Korea; c Natural Products Research Institute, College of Pharmacy, Seoul National University, Seoul, South Korea; d College of Science and Technology, Duksung Women’s University, Seoul, South Korea; INDICASAT

**Keywords:** comparative genomics, genome mining, secondary metabolites, biosynthetic gene clusters

## Abstract

The genus *Streptomyces* is one of the richest sources of secondary metabolite biosynthetic gene clusters (BGCs). Sequencing of a large number of genomes has provided evidence that this well-known bacterial genus still harbors a large number of cryptic BGCs, and their metabolites are yet to be discovered. When taking a gene-first approach for new natural product discovery, BGC prioritization would be the most crucial step for the discovery of novel chemotypes. We hypothesized that strains with a greater number of BGCs would also contain a greater number of silent unique BGCs due to the presence of complex regulatory systems. Based on this hypothesis, we employed a comparative genomics approach to identify a specific *Streptomyces* phylogenetic lineage with the highest and yet-uncharacterized biosynthetic potential. A comparison of BGC abundance and genome size across 158 phylogenetically diverse *Streptomyces* type strains identified that members of the phylogenetic group characterized by the formation of rugose-ornamented spores possess the greatest number of BGCs (average, 50 BGCs) and also the largest genomes (average, 11.5 Mb). The study of genetic and biosynthetic diversities using comparative genomics of 11 sequenced genomes and a genetic similarity network analysis of BGCs suggested that members of this group carry a large number of unique BGCs, the majority of which are cryptic and not associated with any known natural product. We believe that members of this *Streptomyces* phylogenetic group possess a remarkable biosynthetic potential and thus would be a good target for a metabolite characterization study that could lead to the discovery of novel chemotypes.

**IMPORTANCE** It is now well recognized that members of the genus *Streptomyces* still harbor a large number of cryptic BGCs in their genomes, which are mostly silent under laboratory culture conditions. Activation of transcriptionally silent BGCs is technically challenging and thus forms a bottleneck when taking a gene-first approach for the discovery of new natural products. Thus, it is important to focus activation efforts on strains with BGCs that have the potential to produce novel metabolites. The clade-level analysis of biosynthetic diversity could provide insights into the relationship between phylogenetic lineage and biosynthetic diversity. By exploring BGC abundance in relation to *Streptomyces* phylogeny, we identified a specific monophyletic lineage associated with the highest BGC abundance. Then, using a combined analysis of comparative genomics and a genetic network, we demonstrated that members of this lineage are genetically and biosynthetically diverse, contain a large number of cryptic BGCs with novel genotypes, and thus would be a good target for metabolite characterization studies.

## INTRODUCTION

The bacterial genus *Streptomyces* belonging to the phylum *Actinobacteria* is among the largest producers of secondary metabolites. Most importantly, *Streptomyces* spp. have been the major source of clinically important antibiotics, accounting for more than 70% of commercially available antibiotics (1, 2). Examples include the lipopeptide antibiotic daptomycin that is active against multidrug-resistant superbacteria and the aromatic polyketide antibiotic tetracycline that is used against both Gram-positive and Gram-negative bacterial infections (2, 3). Although a large number of metabolites have already been described from this genus over the last century, genome sequencing campaigns conducted for the last decade have demonstrated that chemical diversity captured by the traditional culture-based approach is the tip of the iceberg, and there are still a significant number of cryptic biosynthetic gene clusters (BGCs) present in *Streptomyces* genomes whose metabolites are yet to be discovered (4, 5). Therefore, these cryptic BGCs represent a promising resource for new natural products that could potentially lead to the discovery of next-generation antibiotics.

The genome mining approach for the discovery of new microbial metabolites from cryptic BGCs is composed of two parts: (i) a bioinformatics part being responsible for identifying cryptic BGCs, and (ii) an experimental part being devoted to structural and biological characterization of their metabolites (6–8). As genome sequencing has become a routine task, a number of useful bioinformatics tools have been developed to facilitate the mining of genomes to identify BGCs based on a sequence similarity to those of previously characterized BGCs (9, 10). As a result, the bioinformatics part is nowadays straightforward; however, identification, isolation, and characterization of new metabolites still require a significant amount of time and effort, thus forming a major bottleneck in the overall process (11–15). The major problem is associated with the silencing of BGCs under laboratory culture conditions, probably due to the presence of complex regulatory systems that tightly control the transcriptions of BGCs in response to environmental conditions. As the activation of silent BGCs requires either extensive culture optimization (16, 17) or time-consuming genetic manipulation (18, 19), strain prioritization would be a crucial step in order to focus the time and efforts devoted to activating silent BGCs on strains with a higher potential to result in the discovery of novel chemotypes.

A phylogenetic study of *Streptomyces* could provide insight into species diversity and their grouping based on evolutionary relationships. A comprehensive phylogenetic study was previously conducted by Labeda et al. using full-length 16S rRNA sequences of 615 *Streptomyces* type strains (20). This collaborative study reported the grouping of *Streptomyces* strains into 130 distinct clades based on statistically significant evolutionary relatedness, providing an evolutionary blueprint of the genus *Streptomyces*. Evaluation of biosynthetic diversity at the clade level should thus enable the identification of a *Streptomyces* phylogenetic lineage possessing the greatest biosynthetic potential with a plethora of cryptic silent BGCs. We hypothesized that *Streptomyces* strains with a greater number of BGCs might also contain a greater number of cryptic silent BGCs due to the presence of complex transcriptional regulatory systems that function to avoid coexpression of multiple BGCs that might be harmful to cell growth. Therefore, the *Streptomyces* strains with the greatest number of BGCs would be a good target for a BGC activation study that could lead to the discovery of metabolites with novel chemotypes.

In this study, we attempted to explore the biosynthetic diversity of *Streptomyces* spp. in relation to their 16S rRNA phylogeny with an aim to identify any specific phylogenetic lineage with the greatest hidden biosynthetic potential. To this end, we first constructed a comprehensive 16S rRNA evolutionary tree using *Streptomyces* type strains and analyzed BGC abundance for the strains with sequenced genomes. One monophyletic group characterized by the formation of rugose-ornamented spores was identified to possess the greatest number of BGCs (50 BGCs on average) and also the largest genome (11.5 Mb on average). We further distinguished members of this group into six subgroups based on the 16S rRNA subtree, which was further supported by genome sequence similarity analysis such as whole-genome alignments and pan-genome analysis. The analysis of genetic similarity of all BGCs identified in this group revealed that the majority of BGCs are cryptic and also either subgroup specific (rare) or strain specific (unique). Overall, the result of our analysis suggested that the *Streptomyces* phylogenetic lineage commonly characterized by the rugose-ornamented spores is a rich source of cryptic BGCs with novel genotypes and thus could be a promising target for a metabolite characterization study that could lead to the discovery of metabolites with novel chemotypes.

## RESULTS AND DISCUSSION

### BGC abundance in relation to *Streptomyces* phylogeny.

**FIG 1:**
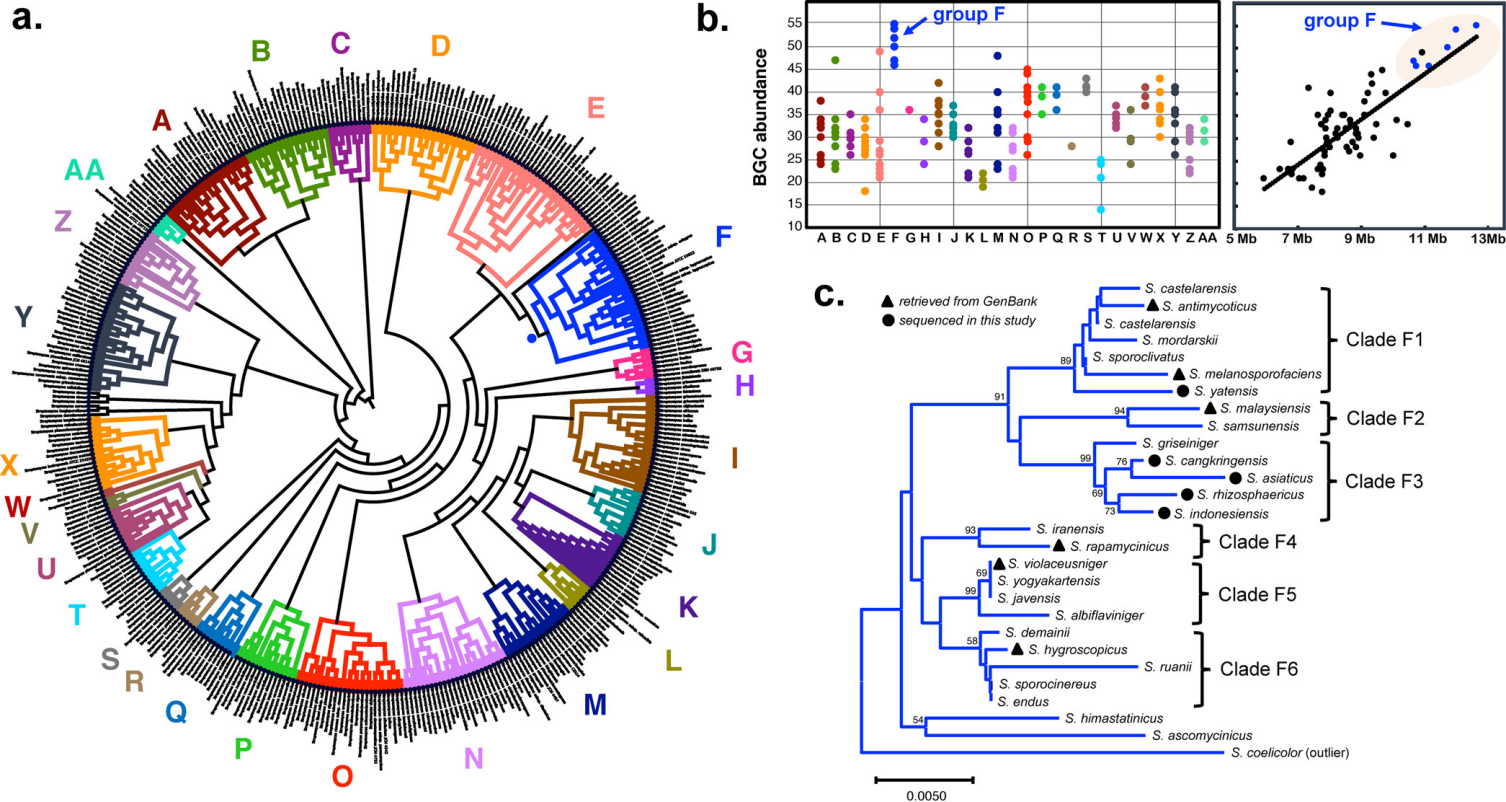
*Streptomyces* 16S rRNA phylogeny and BGC abundance. (a) Neighbor-joining tree of 413 *Streptomyces* 16S rRNA sequences with the Jukes-Cantor distance measure. Bootstrap values were calculated from 1,000 bootstrap replications. Different monophyletic clades are highlighted in different colors. (b) BGC abundance analysis of 152 *Streptomyces* genome sequences. BGC abundance stands for the number of BGCs per genome. BGCs in each genome were predicted by antiSMASH with the detection strictness set to “relaxed.” The graph on the left shows the distribution of BGC abundance in each monophyletic clade. The relationship between genome sizes and BGC abundances is shown on the right. (c) 16S rRNA subtree of the monophyletic group F. Only bootstrap values >50 are shown.

BGC abundance, which is defined here as the number of BGCs in one genome, could be used as a measure of strain-level biosynthetic diversity. To assess the BGC abundance of *Streptomyces* strains in relation to their 16S rRNA phylogeny, we first constructed a comprehensive 16S rRNA evolutionary tree using 410 different *Streptomyces* type strains previously described by Labeda et al. (20). A neighbor-joining tree with 1,000 bootstrap replications was constructed from the alignment of full-length 16S rRNA sequences using the Jukes-Cantor distance measure. The overall tree topology was similar to that of the previously reported tree, with minor discrepancies in clades supported by low bootstrap values (60% to 70%) (Fig. 1a). Next, we evaluated BGC abundance for strains with genome sequences available in the public databases. The draft or complete genome sequences of a total of 292 strains were available in the NCBI genome database among 410 type strains used for the tree construction. Of the 292 sequences, draft genome sequences composed of more than 100 contigs were omitted from the analysis, as a large number of contigs could overestimate BGC abundance due to fragmented BGCs distributed over multiple contigs. A total of 159 complete or draft genome sequences were finally subjected to the BGC abundance analysis using antiSMASH (antibiotics and secondary metabolite analysis shell) (see Data Set S1 in the supplemental material) (21). Although weakly supported (*R*^2^ = 0.63), a linear relationship was observed between genome sizes and BGC abundances (Fig. 1b). The average genome size and BGC abundance were 8.5 Mb and 33 BGCs per genome, respectively. To identify any phylogenetic lineage associated with the greatest BGC abundance, the strains were grouped into monophyletic clades at various branch points, and the distributions of BGC abundance were compared between different groupings. In most of the groupings, a wide distribution of BGC abundance was observed, ranging from 20 to 45 BGCs per genome. However, an exception was apparent for one monophyletic clade that showed an exceptionally high BGC abundance for all the members, which ranged from 45 to 55 BGCs per genome. The strains in this clade also possessed the largest genomes, with a range between 10.7 and 12.7 Mb. Based on a survey of the literature, we found that all members in this monophyletic clade, from hereafter referred to as phylogenetic group F, display a common phenotype represented by the formation of rugose-ornamented spores (22), suggesting the same evolutionary origin.

10.1128/mSystems.00489-21.6 DATA SET S1 Data set used for BGC abundance study. This file contains the list of genomes, accession numbers, and the number of BGCs in each genome and their grouping. Download Data Set S1, XLSX file, 0.1 MB. Copyright © 2021 Chung et al. 2021 Chung et al. https://creativecommons.org/licenses/by/4.0/ This content is distributed under the terms of the Creative Commons Attribution 4.0 International license.

Using the 16S rRNA subtree of the phylogenetic group F (Fig. 1c), we were able to distinguish the group F members into six subgroups, hereafter referred to as subgroups F1 to F6, due to their well-supported bootstrap values. Since the members of this group were expected to have a high biosynthetic potential based on the result of BGC abundance analysis, we decided to perform a detailed analysis of genetic and biosynthetic diversities using high-quality genome sequences. Draft genome sequences were available in the NCBI genome database for six strains, including Streptomyces violaceusniger (subgroup F5), S. melanosporofaciens (subgroup F1), S. castelarensis (subgroup F1), S. malaysiensis (subgroup F2), S. rapamycinicus (subgroup F4), and S. hygroscopicus (subgroup F6). Since there was no sequenced strain for subgroup F3, we decided to sequence the genomes of several members in this subgroup. We also included the strain S. yatensis for genome sequencing in order to increase the sample size of subgroup F1, as it is the largest subgroup. Sequencing of these additional genome sequences should improve the quality of our analysis and thus will provide insights into genetic and biosynthetic diversities of phylogenetic group F at the subclade level.

### Genetic similarity between genomes.

For whole-genome sequencing, the five type strains in group F were obtained from the DSMZ microbial culture collection, including S. rhizosphaericus DSM 41760, S. indonesiensis DSM 41759, S. asiaticus DSM 41761, S. cangkringensis DSM 41769 and S. yatensis DSM 41771. Genomic DNAs were isolated from each strain and sequenced on both PacBio (Pacific Biosciences) and Illumina sequencing platforms to generate long and short sequencing reads. Hierarchical genome assembly (23) using long PacBio and short Illumina sequencing reads resulted in high-quality draft genome sequences consisting of one to six contigs. Since *de novo* assembly of the genomes of *S. yatensis* and *S. indonesiensis* produced only one contig (10.4 Mb) and two contigs (11.5 and 0.2 Mb), respectively, we used these sequences as reference genomes to combine contigs into scaffolds for the other three strains. The contig assembly generated final draft genome sequences consisting of 1 to 3 scaffolds, with the total lengths ranging from 10 to 12 Mb. The sequencing and assembly results are summarized in Table 1. These whole-genome shotgun projects have been deposited at DDBJ/ENA/GenBank (the accession numbers are listed in Table 1). In total, 11 high-quality genome sequences, including 5 newly sequenced genomes (see Fig. S1) and 6 NCBI-derived genome sequences, were used to explore the genetic and biosynthetic diversities of monophyletic group F. All 11 genomes were similar in size (10 to 12 Mb) and BGC abundance (47 to 58 BGCs), confirming that group F members commonly possess large genomes and high BGC abundances compared to those of other *Streptomyces* strains.

**TABLE 1:** Sequencing and assembly statistics of the five *Streptomyces* genomes in group F

Statistic	Data for:				
	Streptomyces yatensis	Streptomyces rhizosphaericus	Streptomyces indonesiensis	Streptomyces asiaticus	Streptomyces cangkringensis
Strain ID	DSM 41771	DSM 41760	DSM 41759	DSM 41761	DSM 41769
Subgroup	F1	F3	F3	F3	F3
NCBI accession no.	CP072941	JAGMTS000000000	JAGSHY000000000	JAGSHX000000000	JAGMTV000000000
Assembly quality	High-quality draft	High-quality draft	High-quality draft	High-quality draft	High-quality draft
Mean subread length (bp) (PacBio)	11,103	8,632	6,940	9,062	7,690
Total no. of subreads (PacBio)	138,664	88,380	175,860	163,203	113,524
Total no. of filtered reads (Illumina)	10,116,770	7,421,758	6,872,284	15,770,942	6,446,662
No. of contigs	1	5	2	6	4
No. of scaffolds	1	3	2	3	2
Longest scaffold (Mb)	10.4	10.5	11.5	11.5	11.5
Estimated genome size (Mb)	10.4	11.0	11.7	11.9	11.7
Genome type	Linear	Linear	Linear	Linear	Linear
*N*_50_ (bp)	10,360,357	9,453,403	11,450,095	10,122,692	9,486,182
Mapped read (%)	99.90	93.42	98.32	99.64	99.65
Depth	88	48	78	78	51
GC (%)	71.4	71.1	71.1	71.0	71.0
CDSs	8,533	9,098	9,645	9,959	9,712
rRNA	6, 6, 6	6, 6, 6	6, 6, 6	6, 6, 6	6, 6, 6
tRNA	88	91	93	91	96
No. of BGCs	47	49	55	54	54

10.1128/mSystems.00489-21.1 FIG S1 Circular maps of 5 newly sequenced genomes. From the outer circle inward, each circle displays information about forward coding DNA sequence (CDS) in dark blue, reverse CDS in dark blue, BGC location in red, tRNA in green, GC plot, and GC skew. The circular genome maps were drawn using DNAPlotter V1.0. Download FIG S1, PDF file, 0.6 MB. Copyright © 2021 Chung et al. 2021 Chung et al. https://creativecommons.org/licenses/by/4.0/ This content is distributed under the terms of the Creative Commons Attribution 4.0 International license.

**FIG 2:**
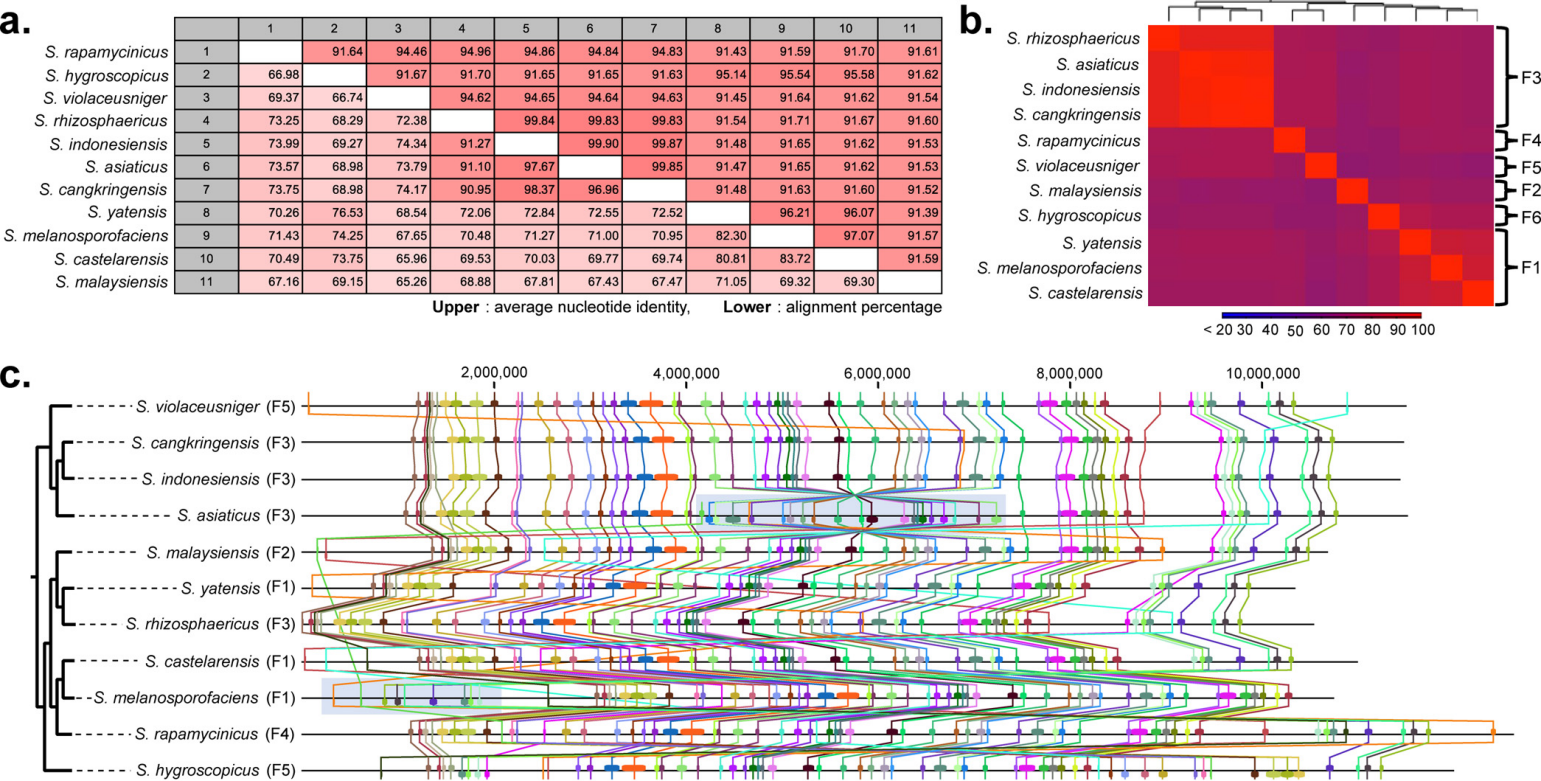
Genome similarity analysis based on the alignment of 11 sequenced genomes in group F. (a) AP and ANI values between genomes. The AP (alignment percentage) value represents the average percentage of aligned genomic regions between two genomes, whereas the ANI (average nucleotide identity) value stands for the percentage of exactly matching nucleotides for these aligned regions. (b) Heat map showing genetic similarity between genomes based on AP values. (c) Genome synteny analysis of 11 sequenced genomes. The colored squares each represent local alignment synteny blocks that are linked together between different genomes by lines with the same colors.

To assess genome similarity between the members of group F, we conducted analyses of sequence similarity and synteny comparison from the alignment of 11 genome sequences with the minimum alignment block set to 100 bp (Fig. 2). The result of sequence similarity analysis was in good agreement with the 16S rRNA phylogeny, as members in the same subgroup showed high average nucleotide identity of aligned region (ANI) and alignment percentage (AP) values. For example, the genomes of *S. rhizosphaericus*, *S. indonesiensis*, *S. asiaticus*, and *S. cangkringensis*, the members of subgroup F3, showed higher than 99% ANI and AP values (Fig. 2a and 2b). A high genome similarity between members in subgroup F3 could be attributed to their geographical origin, as all strains were isolated in close proximity from a forest area inhabited by Paraserianthes falcataria in Java, Indonesia (24), suggesting that members in this clade diverged very recently from the same ancestor. Although these four members in subgroup F3 showed a high similarity of genome sequences, the occurrence of genome rearrangements was observed from the genome synteny analysis. The genome sequence of *S. asiaticus* displayed an inversion of a large genomic region spanning nearly 3 Mb in the center, and also, a deletion of a 0.9-Mb genomic region was observed in one end of the *S. rhizosphaericus* genome (Fig. 2c; see also Fig. S2a). Subgroup F1 members, which include *S. melanosporofaciens*, *S. castelarensis*, and *S. yatensis*, also showed a high similarity in their genome sequences as indicated by high ANI (96 to 97%) and AP (80 to 83%) values, although they were lower than those observed for subgroup F3. In the synteny analysis, these three members displayed similar genome architectures, but transposition of the nearly 2.5-Mb genomic region from one end to the other was seen in the genome of *S. melanosporofaciens* (Fig. 2c; Fig. S2b). The results of synteny analysis suggest that genome rearrangements are a common phenomenon for *Streptomyces*, as frequently observed between strains even with a high sequence similarity, and thus would be one of the major drivers of genetic diversity. For other strains that fall into different subgroups, ANI values ranged from 91% to 96%, and AP values ranged from 60% to 80%; highly variable regions were identified near both ends of the genomes. Overall, the results of genome sequence similarity and synteny analyses led us to the conclusion that significant genetic variations exist between members in different subgroups, but genetic variation is relatively low between members that fall into the same subgroup.

10.1128/mSystems.00489-21.2 FIG S2 Genome alignment results for subgroups F3 (a) and F1 (b). The colored squares represent local alignment synteny blocks that are linked together using same-color lines. The inversion of a large genomic region was observed in the center of the *S. asiaticus* genome. In addition, the genome of *S. rhizosphaericus* showed a deletion of a large genomic region in the front. Download FIG S2, PDF file, 0.1 MB. Copyright © 2021 Chung et al. 2021 Chung et al. https://creativecommons.org/licenses/by/4.0/ This content is distributed under the terms of the Creative Commons Attribution 4.0 International license.

### Pan-genome analysis.

**FIG 3:**
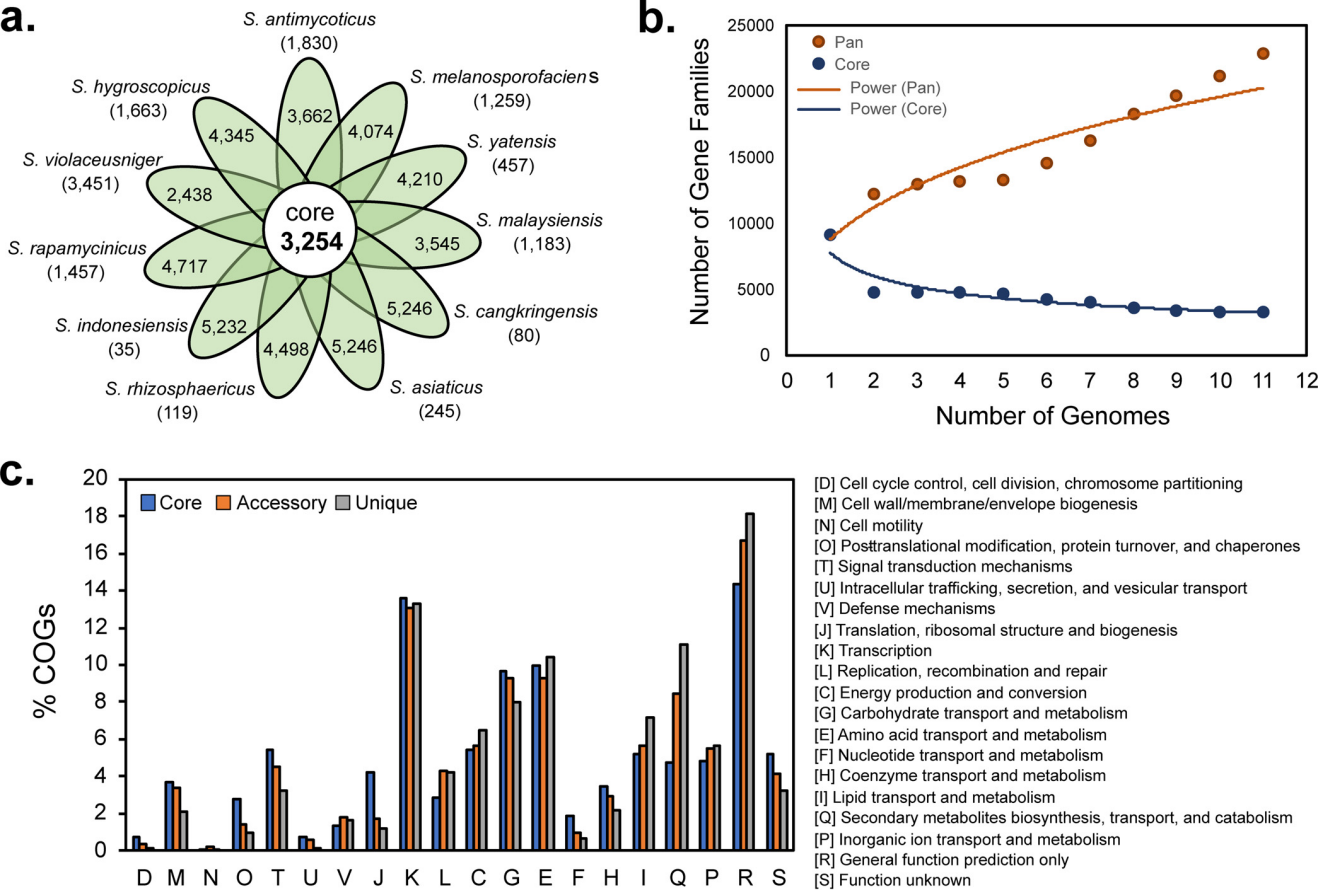
Pan-genome analysis of 11 sequenced genomes in group F. (a) Flower diagram representing the core, accessory, and unique genomes of the 11 sequenced genomes in group F. The numbers in parentheses represent the number of unique genes in each genome. (b) Core/pan-genome plot for group F calculated over 50 iterations using the BPGA analysis tool. Pan-genome is considered to be open. (c) COG functional analysis of pan-genome. The graph shows the predicted function of proteins encoded by core, accessory, and unique genes of the pan-genome.

The combined analysis of genetic and biosynthetic diversities might provide insight into how the *Streptomyces* strains should be prioritized to discover novel chemotypes without redundancy of experiments when mining genomes for new natural products discovery. To obtain additional information about the genetic diversity present in group F, we performed a pan-genome analysis of the 11 sequenced genomes using the BPGA (bacterial pan-genome analysis) pipeline tool (25). This analysis revealed a pan-genome of 22,885 genes composed of a core genome of 3,254 genes, an accessory genome of 7,852 genes, and a unique genome of 11,779 genes (Fig. 3a). The core-pan plot suggested the pan-genome to be open; therefore, the size of the pan-genome would be expanded if more genome sequences from group F are added to the analysis, while the size of the core genome would remain relatively constant (Fig. 3b). The pan-genome analysis predicted that only a small number of unique genes are present in the genomes of *S. cangkringensis* (80 genes), *S. asiaticus* (245 genes), *S. rhizosphaericus* (119 genes), and *S. indonesiensis* (35 genes), which are all members of subgroup F3. In contrast, the genomes of members of subgroup F1 contain a larger number of unique genes, as predicted for S. antimycoticus (1,830 genes), *S. melanosporofaciens* (1,259 genes), and *S. yatensis* (457 genes), indicating that the genetic diversity is higher in subgroup F1 than in F3. The strains *S. malaysiensis* (subgroup F2), *S. rapamycinicus* (subgroup F4), *S. violaceusniger* (subgroup F5), and *S. hygroscopicus* (subgroup F6), which were the only sequenced members in each subgroup, were predicted to harbor 1,183, 1,457, 3,451, and 1,663 unique genes in their genomes, respectively. The largest number of unique genes (3,451) and the smallest number of rare genes predicted to be present in the genome of *S. violaceusniger* might imply that this strain has the most distant evolutional relationship from other members in group F. In a COG (clusters of orthologous groups of proteins) analysis of the pan-genome (26), the core, accessory, and unique genes were distributed across all COG categories (Fig. 3c). Of the COG categories, the large portion of genes (13% of pan-genome) were devoted to the function categorized as “K” (transcription) that includes genes encoding RNA polymerases, sigma factors, and transcriptional regulators and are distributed similarly among core, accessory, and unique genes. As could be expected, this suggests that *Streptomyces* strains possess strain-specific transcriptional regulatory systems that independently regulate the transcription of genes for strain-specific functions, such as the production of secondary metabolites. Since more than 40% of unique genes fell into the three categories, “R” (18%, generation function prediction only), “K” (13.3%, transcription), and “Q” (11.1%, secondary metabolite biosynthesis, transport, and catabolism), genes involved in these functions would be the major contributors to the genetic diversity observed in group F. The genes categorized as “Q” (secondary metabolites biosynthesis, transport, and catabolism) accounted for 8.4% and 11.1% of accessory and unique genes, respectively, whereas only 4.7% of core genes were classified into this category. Based on this result, it could be expected that a significant number of BGCs in the monophyletic group F would be subgroup specific (accessory) or strain specific (unique).

### Biosynthetic diversity.

**FIG 4:**
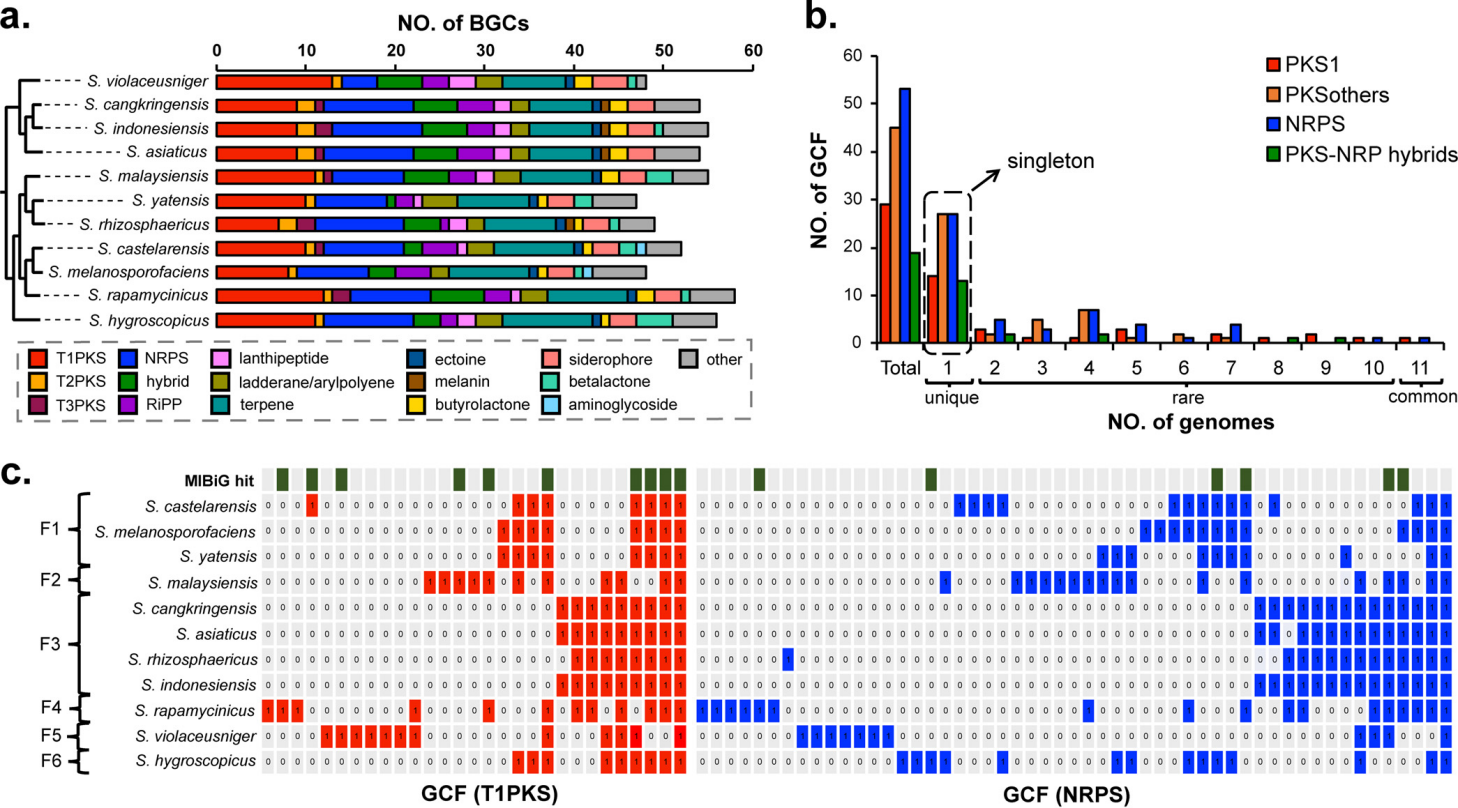
Biosynthetic diversity analysis of monophyletic group F. (a) Comparison of BGC distribution across different BGC families. BGCs identified from antiSMASH analysis were reclassified according to our classification criteria. BGC color codes were maintained as identical throughout the rest of figures. (b) Prevalence analysis of GCFs across 11 genomes. Unique GCFs are only present in one genome, whereas common GCFs are present in all 11 genomes; otherwise, they are rare GCFs. (c) GCF absence/presence analysis. The presence of a GCF is highlighted in red for T1PKS GCFs and blue for NRPS GCFs. GCF clustered with known BGCs from MiBiG database are highlighted in green at the top.

**FIG 5:**
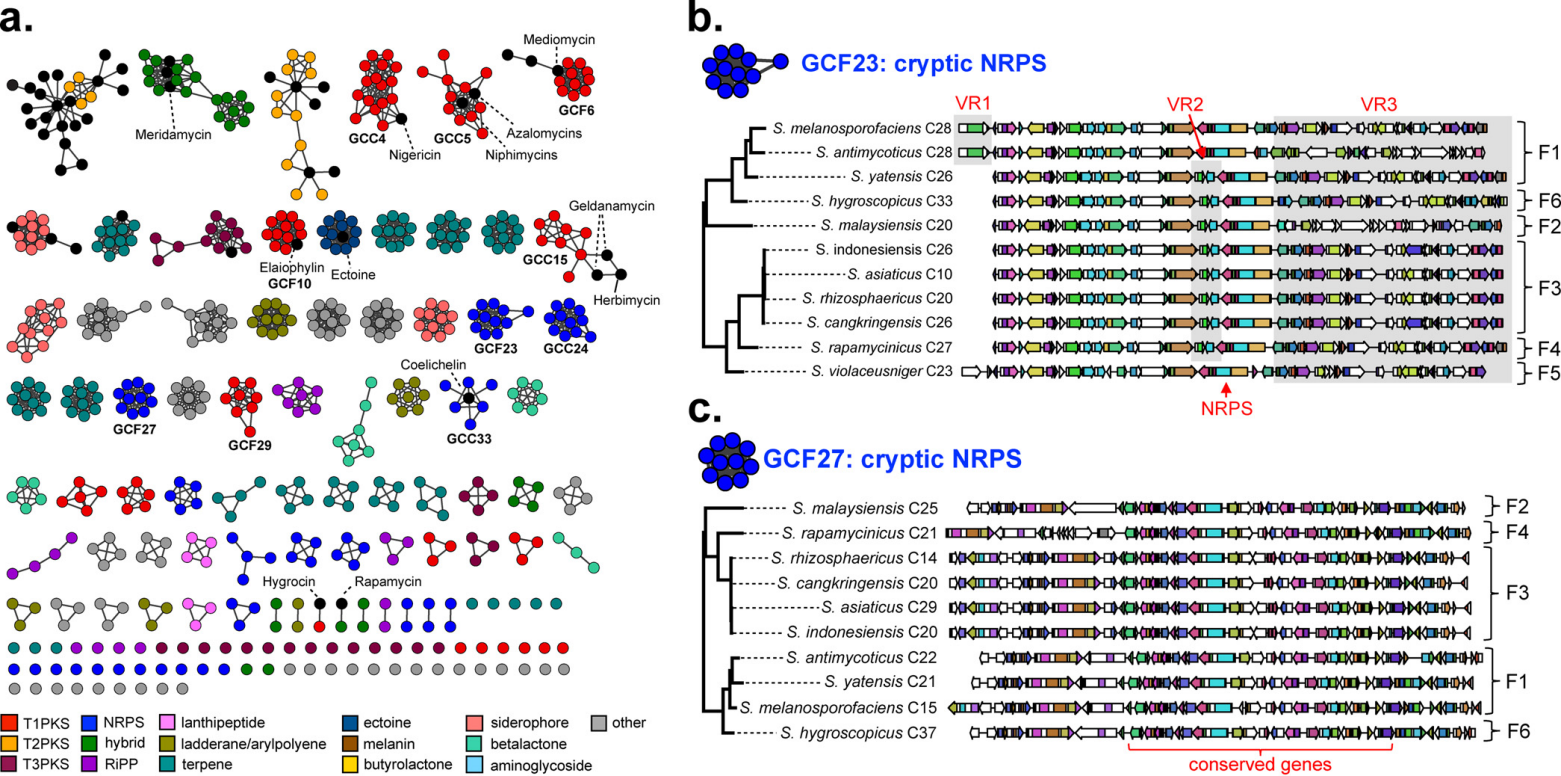
Genetic network analysis and genotype comparison of BGCs. (a) Nodes represent specific BGCs, and the length of edges represents their genetic relatedness based on the Jaccard index values calculated using BiG-SCAPE. The genetic network map of BGCs was visualized on Cytoscape version 3.8.2. The same color code for each BGC type was used as in Fig. 4. (b) BGC genotype comparison for the common GCF cluster GCF-23. BGCs in this cluster are cryptic and not associated with any known BGC. Three variable regions, designated VR1, VR2, and VR3, are highlighted in gray. (c) BGC genotype comparison for the rare GCF cluster GCF-27. BGCs in this cluster are also predicted to be cryptic.

To assess the biosynthetic diversity present in group F, we next performed genetic similarity network analysis of BGCs using the bioinformatics tools antiSMASH and BiG-SCAPE (Fig. 4 and 5). The BiG-SCAPE algorithm generates a BGC similarity network based on clustering using three metrics: the Jaccard index, an adjacency index, and domain sequence similarity (27). For an accurate analysis, only type I polyketide (PK) and nonribosomal peptide (NRP) and their hybrid BGCs were included, as their modular natures give rise to a highly predictable relationship between domain organizations and chemical structures. For other BGC types such as type II PK, ribosomal peptide, and terpene BGCs, genetic similarity networks based on the domain organization of BGCs might overestimate their biosynthetic diversities, because BGCs with a different domain order and low sequence similarity, but the same set of biosynthetic genes, could produce the same metabolites. The total number of BGCs identified by antiSMASH across 11 different genomes was 576 (on average, 52 BGCs per genome) including 109 T1PKS, 96 NRPS, and 44 hybrid gene clusters. All members of group F harbor a remarkably large number of T1PKS and NRPS gene clusters: on average, 10 T1PKS and 9 NRPS gene clusters per genome (Fig. 4a). The members of group F also carry on average 4 PKS-NRPS hybrid gene clusters in their genomes. Among the members, the strain *S. violaceusniger*, a member of subgroup F5, was identified to harbor the largest number of T1PKS but the lowest number of NRPS gene clusters: 13 T1PKS and 4 NRPS gene clusters. Together with the result of the pan-genome analysis, we can expect that the strain *S. violaceusniger* is evolutionarily the most distinct member from other members in group F.

Clustering of BGCs using the BiG-SCAPE algorithm grouped 109 T1PKS gene clusters into 29 gene cluster families (GCFs), 96 NRPS gene clusters into 53 GCFs, and 44 PKS-NRPS hybrid gene clusters into 19 GCFs (Fig. 4b). The larger number of GCFs estimated for NRPS gene clusters than for those of T1PKS reflects the presence of a higher diversity for NRP biosynthesis in group F. The presence or absence of GCFs in each genome in group F is shown in Fig. 4c and summarized in Data Set S2. Only one GCF was present in all 11 genomes for both T1PKS and NRPS clusters, which also formed tight single clusters in the genetic similarity network analysis. The result of BGC clustering also showed that members of subgroup F3 share the same sets of T1PKS and NRPS GCFs, with minor discrepancy (Fig. 4c). However, a significant discrepancy in the distributions of NRPS GCFs was apparent between members in subgroup F1, although they share nearly identical sets of T1PKS GCFs. The largest portion of GCFs appeared to be singletons, meaning that these GCFs are strain specific. The prevalence of GCFs across the members of group F might represent time points when horizontal transfer events occurred in evolutionary history (see Fig. S3). Based on the prevalence pattern, we classified GCFs into common GCFs if found across all subgroups, rare GCFs if found in more than two subgroups but not in all, and unique GCFs if found in only one subgroup. As more than half of all GCFs were unique GCFs, it is probably reasonable to say that the remarkable biosynthetic diversity observed in group F could be attributed to horizontal transfer events that occurred at relatively recent time points in their evolutionary history instead of genetic diversification through a vertical transfer of BGCs.

10.1128/mSystems.00489-21.3 FIG S3 Evolutionary subtree of group F members. The colored boxes represent possible timepoints when horizontal transfer events occurred for GCFs. Common GCFs are present across all members in group F; thus, these GCFs were likely to have been acquired long before the common ancestor divided into any sublineages. Rare GCFs are present in more than two subgroups but not in all. These GCFs would have been acquired by horizontal transfer events after the common ancestor of group F divided into several sublineages. Unique GCFs are only present in one subgroup or are strain specific. Therefore, these GCFs are likely to have been acquired by very recent horizontal events after the final six sublineages were formed. Download FIG S3, PDF file, 0.03 MB. Copyright © 2021 Chung et al. 2021 Chung et al. https://creativecommons.org/licenses/by/4.0/ This content is distributed under the terms of the Creative Commons Attribution 4.0 International license.

10.1128/mSystems.00489-21.7 DATA SET S2 Data set generated by BiG-SCAPE algorithm. This file contains the list of GCF families for PKS, NRPS, and PKS-NRPS hybrid BGC types. Download Data Set S2, XLSX file, 0.1 MB. Copyright © 2021 Chung et al. 2021 Chung et al. https://creativecommons.org/licenses/by/4.0/ This content is distributed under the terms of the Creative Commons Attribution 4.0 International license.

### Genotype variation between BGCs.

To further evaluate the BGC diversity of group F, we next assessed evolutionary relationships between BGCs in the same GCF based on their domain compositions and synteny. BGC evolutionary trees were constructed using the CORASON (core analysis of synthetic orthologues to prioritize natural product biosynthetic gene clusters) pipeline implemented in the BiG-SCAPE analysis. To allow for genotype-based dereplication of BGCs, we also included known BGCs deposited in the latest MiBiG database (version 2.0, total 1,923 clusters) (28, 29) in the BiG-SCAPE analysis. GCFs that were further clustered into clans in the BiG-SCAPE analysis were merged after manual inspection and designated gene cluster clans (GCCs) as summarized in Data Set S3. We first assessed genotype variations between BGCs for common T1PKS and NRPS GCFs. The topologies of both common T1PKS and NRPS GCF evolutionary trees were in good agreement with that of the 16S rRNA tree with the same subgrouping, indicating that BGCs in common GCFs were vertically transferred through phylogenetic lineages. In the network analysis, the common T1PKS GCF referred to as GCF-10 also clustered with the known elaiophylin BGC (Fig. 5a). The set of genes and domains required for elaiophylin biosynthesis as well as their synteny was well conserved across all 11 strains, whereas small variations were seen outside the BGCs (see Fig. S4a). After the first report of the isolation of elaiophylin, a 16-membered macrolide, in 1959 from *S. melanosporofaciens* (30), the isolation of the same compound or its analogs has been reported from several *Streptomyces* species, such as *S. hygroscopicus* and *S. melanosporofaciens* (31), and they all seem to belong to the monophyletic group F. The NRPS GCF-23 was another common GCF present across all members of group F. GCF-23 showed no association with any known BGC deposited in the MiBiG database; thus, BGCs in this GCF are likely to be cryptic. Although the core set of biosynthetic genes, including the single domain NRPS gene, is well conserved across BGCs, some variations were also apparent (Fig. 5b). For example, the two genes upstream of the NRPS gene (VR2 [variable region 2]) that are predicted by BLAST to encode YqcI/YclG family protein and polysaccharide deacetylase were absent in some members. Also, the region downstream of the NRPS gene is somewhat variable between BGCs in different subgroups. Since most of the genes in the variable regions were not predicted to be biosynthetic genes by BLAST, it is not certain that any chemotype variation would exist between their metabolites. Taken together, the comparison of BGC genotypes for common T1PKS and NRPS GCFs suggests that the BGC genotypes have been well conserved throughout the evolutionary period without significant diversification; thus, it could be inferred that metabolites encoded by these GCFs would be hallmarks of the monophyletic group F and are likely to have played an important role in the survival of this phylogenetic lineage.

10.1128/mSystems.00489-21.4 FIG S4 Genotype comparison between BGCs for GCF-10 and GCC-4. The evolutionary tree was constructed using the CORASON pipeline implemented in the BIG-SCAPE analysis. (a) The common T1PKS GCF-10 network cluster was associated with the known elaiophylin BGC. (b) Two separate GCFs assigned by BIG-SCAPE analysis were connected to form the common GCC-4 cluster in the GCF network analysis. This common T1PKS network cluster was also associated with the known nigericin BGC. Download FIG S4, PDF file, 0.3 MB. Copyright © 2021 Chung et al. 2021 Chung et al. https://creativecommons.org/licenses/by/4.0/ This content is distributed under the terms of the Creative Commons Attribution 4.0 International license.

10.1128/mSystems.00489-21.8 DATA SET S3 Data set of BGC network clustering. This file contains the list of all BGCs for each member of the phylogenetic group F and their GCF assignments. The result of BGC network clustering is summarized in the first sheet. Download Data Set S3, XLSX file, 0.1 MB. Copyright © 2021 Chung et al. 2021 Chung et al. https://creativecommons.org/licenses/by/4.0/ This content is distributed under the terms of the Creative Commons Attribution 4.0 International license.

In the network analysis, clusters of several rare GCFs were connected together, forming common GCC network clusters, designated GCC-4 and GCC-5 (Fig. 5a). The network cluster GCC-4 was also linked to the previously characterized nigericin BGC (32), whereas the network cluster GCC-5 was associated with the known niphimycin (33) and azalomycin (34) BGCs. The comparison of genotypes between BGCs in GCC-4 revealed some variations, especially between BGCs belonging to different subgroups (Fig. S4b). A prominent difference was the insertion of a number of genes between the two modular T1PKS gene operons as observed for BGCs in subgroups F3 and F4 (highlighted in gray in the supplemental figure). These additional genes were predicted by BLAST to encode regulatory and transport proteins. However, it needs to be determined if metabolites produced by these BGCs display different chemotypes, as the domain organizations of core T1PKS genes seem to be well conserved, but it was difficult to estimate the difference in precursor specificity for each T1PKS module. In the case of GCC-5, variations in BGC genotypes were obvious even for core T1PKS genes, with each displaying small differences in domain and module organizations (see Fig. S5a). This suggests that BGCs in GCC-5 would produce metabolites with similar but different chemotypes. Other rare GCF clusters, GCF-6 (T1PKS) and GCF-27 (NRPS), were only missing in the genome of *S. violaceusniger*, the member of subgroup F5 (Fig. 5c and Fig. S5b). GCF-6 was associated with the previously characterized mediomycin (35) and ECO-02301 (36) BGCs, whereas GCF-27 was cryptic. The GCF-6 cluster showed genotype variations in both core T1PKS and tailoring genes; thus, it is expected that metabolite characterization studies of BGCs in GCF-6 would lead to the discovery of a series of structural analogs of mediomycin. Based on the overall results of genotype comparison, we were able to conclude that BGC genotypes were highly conserved for common GCFs/GCCs, but variations were frequently observed between BGCs for rare GCFs/GCCs, especially at the subgroup level; therefore, the metabolite characterization of BGCs in rare GCFs/GCCs would be a good strategy to identify a series of structural congeners.

10.1128/mSystems.00489-21.5 FIG S5 Genotype comparison between BGCs for GCC-5 and GCF-6. The evolutionary tree was constructed using the CORASON pipeline implemented to the BIG-SCAPE analysis. (a) The common T1PKS network cluster GCC-5 was associated with the known azalomycin and niphimycin BGCs. (b) The rare T1PKS GCF cluster GCF-6 was connected to the known mediomycin and ECO-02301 BGCs in the GCF network analysis. Slight variations in T1PKS module organization were observed between BGCs. Download FIG S5, PDF file, 0.4 MB. Copyright © 2021 Chung et al. 2021 Chung et al. https://creativecommons.org/licenses/by/4.0/ This content is distributed under the terms of the Creative Commons Attribution 4.0 International license.

Last, we turned our attention to unique GCFs, as they are likely to be major contributors to the overall biosynthetic diversity in group F. The genetic network showing an all-versus-all comparison of BGCs revealed the presence of a total of 89 singletons across 11 different genomes. Among the singletons was the rapamycin BGC in the genome of *S. rapamycinicus*, the only known rapamycin producer (formally classified as *S. hygroscopicus*) (37). The rare nature of the rapamycin BGC explains why the isolation of rapamycin and its congeners has not been reported from other sources after the first isolation of rapamycin in 1975 (38). GCF-67 was another unique GCF and clustered with the previously known hygrocin BGC from the MiBiG database. The manual inspection of this cluster suggested that GCF-67 could be merged with GCC-15, potentially creating an additional common GCF cluster. The separate clustering was due to variations in gene orders and contents between BGCs in GCF-67 and GCC-15. The common nature of hygrocin BGC (39) is also supported by the fact that hygrocins and their congeners, the ansamycin family of polyketides, have been isolated from diverse *Streptomyces* strains (40). Except for these two GCFs, the rest of the unique GCFs were cryptic, being not associated with any known BGC from the MiBiG database, and thus are expected to produce metabolites with novel chemotypes.

Based on the genetic network analysis of BGCs, we were able to reach the following conclusions about the biosynthetic potential of the members of *Streptomyces* monophyletic group F. First, GCF types that were most associated with known BGCs were the common T1PKS GCFs as exemplified by GCC-4, GCC-5, and GCF-10. Second, except for GCC-33, which was associated with the known siderophore coelichelin BGC, the vast majority of NRPS GCF network clusters were not associated with any known BGC, suggesting that metabolites of NRPS BGCs have been significantly undercharacterized in spite of their high biosynthetic potential (total of 96 NRPS BGCs clustered into 53 GCFs, as shown in Fig. 4c): for example, even the common NRPS GCF cluster GCF-23 is cryptic. Third, rare and unique GCFs that are either subgroup specific or strain specific were mostly cryptic. These overall results suggest that all NRPS gene clusters and rare and unique T1PKS and T1PKS-NRPS hybrid gene clusters should be good targets for a metabolite characterization study that could lead to the discovery of metabolites with novel chemotypes.

### Conclusion.

It is now well accepted that cryptic BGCs are prevalent in *Streptomyces* genomes, outnumbering previously characterized BGCs. Although the search for cryptic BGCs from sequenced genomes is quite straightforward due to publicly available bioinformatics tools specialized for BGC searches, the characterization of their metabolites still requires significant experimental efforts, because the majority of BGCs are transcriptionally silent under laboratory culture conditions. Therefore, it is important to focus metabolite characterization efforts on BGCs that are likely to generate novel chemotypes. Here, we hypothesized that *Streptomyces* strains with a greater number of BGCs would contain a greater number of silent and unique BGCs that could lead to the discovery of novel metabolites and therefore would be a good target for a genomics-based approach for the discovery of new natural products.

To gain insights into the biosynthetic potential of *Streptomyces* in relation to their phylogeny, we constructed the 16S rRNA phylogeny using 410 phylogenetically diverse *Streptomyces* type strains and surveyed BGC abundance using 159 high-quality complete or draft genome sequences. This comprehensive analysis allowed us to identify the particular monophyletic clade (group F) that was morphologically characterized by the formation of rugose-ornamented spores and genetically characterized by the largest genomes and greatest number of BGCs (on average, 11.5 Mb genome size and 50 BGCs per genome). Based on the 16S rRNA subtree, the members of group F were further distinguished into six subgroups (F1 to F6). The genetic and biosynthetic diversity study of group F using 11 genome sequences, of which 5 genomes were newly sequenced and 6 genome sequences were derived from the public database, suggested that significant genetic and biosynthetic variations exist between members in different subgroups, whereas members within the same subgroup are similar in their genomes, with minor variations. Based on the result of a pan-genome analysis, genes related to transcriptional regulatory systems and secondary metabolite biosynthesis are expected to be major contributors to the genetic and biosynthetic diversities of group F. The remarkable biosynthetic potential of group F could be inferred from the presence of 109 T1PKS, 96 NRPS, and 44 T1PKS-NRPS hybrid gene clusters across 11 genomes that could be grouped into 29, 53, and 19 GCFs, respectively. Higher diversity was seen for NRPS BGCs than for T1PKS clusters. In addition, T1PKS GCFs/GCCs were more frequently associated with known BGCs, whereas the majority of NRPS GCFs/GCCs were cryptic. The important observation was that the unique GCFs are the major contributors to the overall biosynthetic diversity seen in group F and are also mostly cryptic regardless of their BGC types. This suggests that unique GCFs that have been acquired by recent horizontal transfer events would be a good target for a metabolite characterization study that could lead to the discovery of novel chemotypes.

As exemplified in this paper, the analysis of biosynthetic diversity in relation to the 16S rRNA phylogeny could provide valuable information about how to prioritize strains and BGCs to discover metabolites with novel chemotypes using a genomics-based approach. As part of our ongoing efforts to discover novel biologically active compounds from nature, we are now in the process of systematic cloning and genetic refactoring-based activation of the unique cryptic BGCs present in members of this biosynthetically talented *Streptomyces* monophyletic group.

## MATERIALS AND METHODS

### 16S rRNA phylogeny and BGC abundance analysis.

The complete 16S rRNA gene sequences of the *Streptomyces* type strains were retrieved from the National Center for Biotechnology Information (NCBI) database using the accession numbers reported by Labeda et al. (20). The 413 sequences were aligned using CLC Main Workbench (Qiagen) with default settings. The resulting sequence alignment was used for the construction of the phylogenetic tree in MEGA X (41). The evolutionary relationship was inferred using the neighbor-joining method with the Jukes-Cantor distance measure and 1,000 bootstrap replications. The Escherichia coli 16S rRNA sequence was used as an outlier. The tree was visualized using Interactive Tree Of Life (iTOL) version 3 (42). The subtree of the rugose-ornamented spore clade was constructed in the same way as described above.

The BGC abundance was defined as the number of BGCs per genome. Of the 413 type strains used for the 16S rRNA phylogeny, the genome sequences of 292 strains were available in the public databases. To improve the accuracy of our analysis, complete sequences or draft genome sequences consisting of less than 100 contigs were retrieved from the NCBI database, comprising the final set of 158 genome sequences. The annotated genome sequences in the GenBank file format were used for the BGC abundance analysis using antiSMASH 5.0 (antibiotics and secondary metabolite analysis shell). The “relaxed” detection strictness was used for all analyses. For the draft genome sequences with multiple contigs, we manually analyzed the antiSMASH result to avoid any possible duplication in counting the number of BGCs.

### Culture condition and gDNA isolation.

Of the strains in group F, genome sequences of six strains were retrieved from the NCBI database. To increase the sample size of our genetic and biosynthetic diversity analysis, we sequenced the whole genomes of five additional strains in group F. Strains of *S. rhizosphaericus* (DSM 41760), *S. indonesiensis* (DSM 41759), *S. asiaticus* (DSM 41761), *S. cangkringensis* (DSM 41769), and *S. yatensis* (DSM 41771) were obtained from the DSMZ culture collection (German Collection of Microorganisms and Cell Cultures GmbH) and maintained on ISP4 agar plates. For the isolation of genomic DNAs (gDNAs), the five strains were cultured in the tryptic soy broth (TSB) liquid medium for 3 days, and high-quality gDNAs for whole-genome sequencing were isolated using the previously described salting-out method (43).

### Whole-genome sequencing.

Sequencing of the five genomes was performed on both PacBio and Illumina platforms (Macrogen). For PacBio sequencing, sequencing templates were prepared using the SMRTbell template prep kit according to the guide and sequenced on the PacBio RS system. In the case of Illumina sequencing, sequencing libraries were prepared by random fragmentation of genomic DNAs, followed by end-repair and ligation with the sequencing adapter on both ends. The prepared libraries were loaded onto the flow cells where the libraries were captured on surface-bound oligonucleotides complementary to the library adapters. Library fragments were amplified into clonal clusters via bridge amplification and sequenced using Illumina sequencing by synthesis (SBS) technology. *De novo* assembly of genomic DNA sequences was performed using both long reads generated by PacBio sequencing and short reads generated by Illumina sequencing. First, preassembly was carried out by mapping single-pass long reads to seed reads (the longest portion in the read length distribution), and then the assembly was performed using HGAP3 (Hierarchical Genome Assembly Process 3) (44). After the assembly, the Illumina short reads were aligned to the PacBio draft assembly for error correction using the Pilon error correction tool (45). The *de novo* assembly process generated one to six high-quality contigs for the five genomes. Lastly, since only one contig (10.4 Mb) was obtained from the *de novo* assembly, we used the genome sequence of *S. yatensis* as a reference to further assemble contigs into scaffolds for other genomes with multiple contigs. The contigs were aligned to the genome sequence of *S. yatensis* using the Qiagen CLC genome finishing module and merged into scaffolds, resulting in the final three scaffolds for the *S. rhizosphaericus* genome (10.5 Mb, 0.4 Mb, and 0.1 Mb), two scaffolds for *S. indonesiensis* (11.5 Mb and 0.2 Mb), three scaffolds for *S. asiaticus* (11.5 Mb, 0.2 Mb, and 0.1 Mb), and two scaffolds for *S. cangkringensis* (11.5 Mb and 0.2 Mb). Annotation of the five resulting draft genome sequences was carried out using the RAST server as well as GeneMark (46, 47).

### Whole-genome alignment and pan-genome analysis.

The alignment of 11 genome sequences was performed using the whole-genome alignment plugin of the CLC Genomics Workbench (Qiagen). Briefly, short seed sequences (minimum initial seed length, 15 bp) that were shared between genomes were identified and extended using a HOXD scoring matrix until the local alignment score dropped below a fixed threshold (48). Distance matrixes between genome sequences were calculated from the initial extended seed matches and used for the subsequent pairwise processing, in which the most similar genomes were processed first. Proceeding iteratively on the most similar genome pair, the seed matches were extended and merged to create longer alignment blocks (minimum alignment block length, 100 bp). From the result of the whole-genome alignment, the genome similarity between two genomes was evaluated using AP (alignment percentage) and ANI (average nucleotide identity) measures. The AP value represents the average percentage of aligned genomic regions between two genomes, whereas the ANI value stands for the percentage of exactly matching nucleotides in the aligned regions. These values provide a quantitative measure of the similarity between two genomes. Then, the similarity matrix from the pairwise comparison table was used to create a heat map using the specified clustering options (Euclidean distance measure and average linkage criteria) and a hierarchical clustering algorithm.

Core/pan-genome analysis was performed using the BPGA (Bacterial Pan Genome Analysis) tool and 11 annotated genome sequences in GenBank formats (25). The USEARCH algorithm with the similarity threshold set to 0.5 was used to identify orthologous genes. No significant difference was observed in the result when the threshold value was changed to 0.3, 0.4, 0.6, or 0.7. Core genome and pan-genome plots were generated by 500 iterative calculations.

### Genetic similarity network analysis of BGCs.

The annotated 11 whole-genome sequences in the GenBank file format were subjected to BGC identification using the genome mining pipeline antiSMASH 5.0 (21). The detection strictness was set to “relaxed,” and the ClusterBlast and cluster comparison options were turned on for the antiSMASH analysis. For each BGC, a BGC type assigned by antiSMASH was manually inspected and reclassified according to our classification criteria. The resulting 576 putative BGCs across 11 genome sequences from the monophyletic group F as well as 1,923 previously characterized BGCs from the MiBiG (Minimum Information about a Biosynthetic Gene Cluster) database (version 2.0, October 2019) were subjected to the genetic similarity network analysis using the BiG-SCAPE (biosynthetic genes similarity clustering and prospecting engine) pipeline (27–29). The resulting sequence similarity network with a default similarity score cutoff (c = 0.3; threshold of 30%) consisted of 609 unique nodes and 1,655 edges. Obtained sequence similarity matrices were then visualized using Cytoscape 3.6.1 (49).

### Data availability.

The *S. yatensis* whole-genome shotgun project has been deposited at DDBJ/ENA/GenBank under the accession number CP072941, BioSample accession SAMN18614723, and BioProject accession PRJNA719702. The *S. rhizosphaericus* whole-genome shotgun project has been deposited at DDBJ/ENA/GenBank under the accession number JAGMTS000000000, BioSample SAMN18628702, and BioProject PRJNA719933. The version described in this paper is version JAGMTS010000000. The *S. indonesiensis* whole-genome shotgun project has been deposited at DDBJ/ENA/GenBank under the accession number JAGSHY000000000, BioSample SAMN18644739, and BioProject PRJNA719933. The version described in this paper is version JAGSHY010000000. The *S. asiaticus* whole-genome shotgun project has been deposited at DDBJ/ENA/GenBank under the accession number JAGSHX000000000, BioSample SAMN18644713, and BioProject PRJNA719933. The version described in this paper is version JAGSHX010000000. The *S. cangkringensis* whole-genome shotgun project has been deposited at DDBJ/ENA/GenBank under the accession number JAGMTV000000000, BioSample SAMN18644715, and BioProject PRJNA719933. The version described in this paper is version JAGMTV010000000.
