# Supplementary figures and images for: Comparative Genomics Reveals a Remarkable Biosynthetic Potential of the Streptomyces Phylogenetic Lineage Associated with Rugose-Ornamented Spores
Source: mSystems. 2021 Aug 24;6(4):e00489-21. doi: 10.1128/mSystems.00489-21 (PMC8407293; doi:10.1128/mSystems.00489-21)

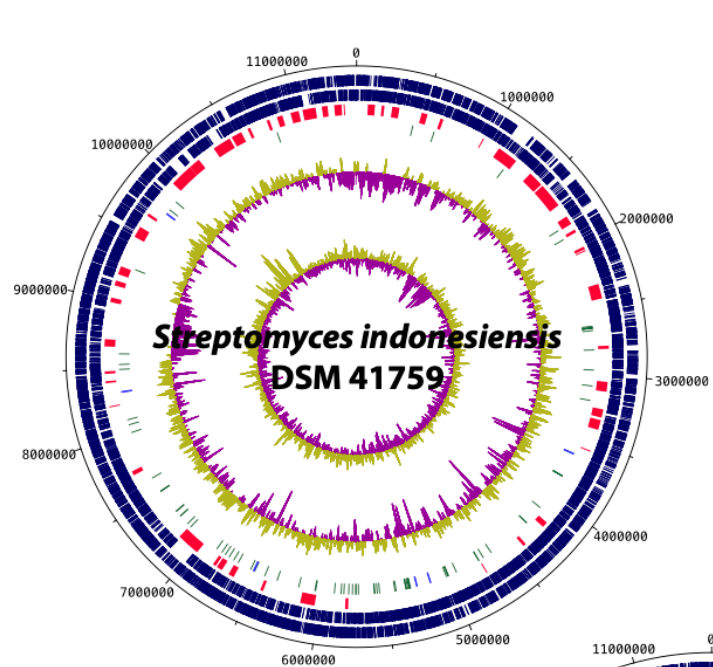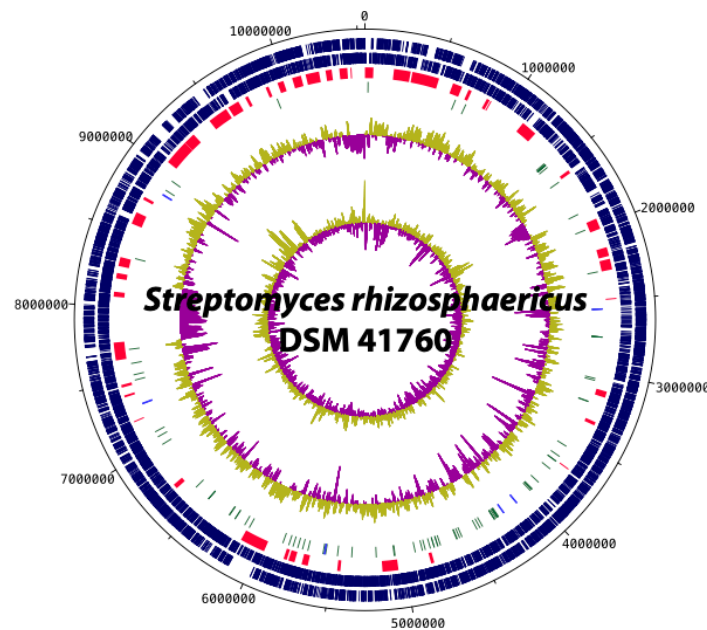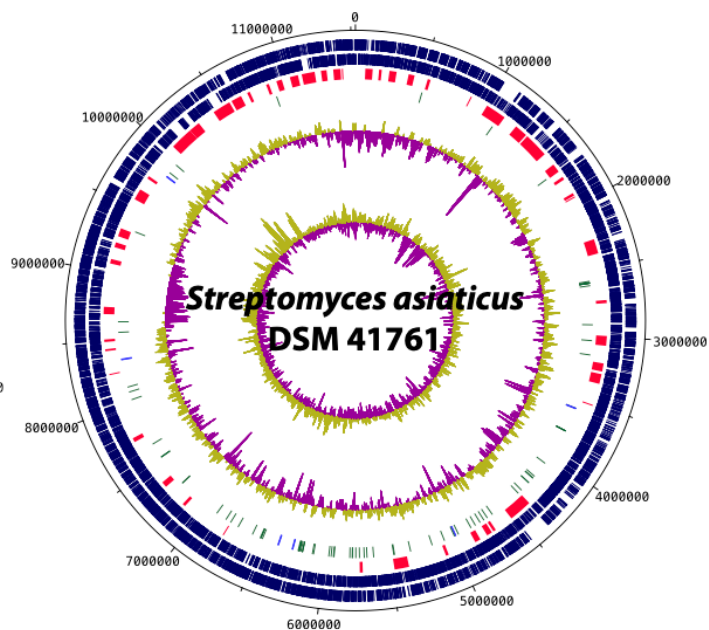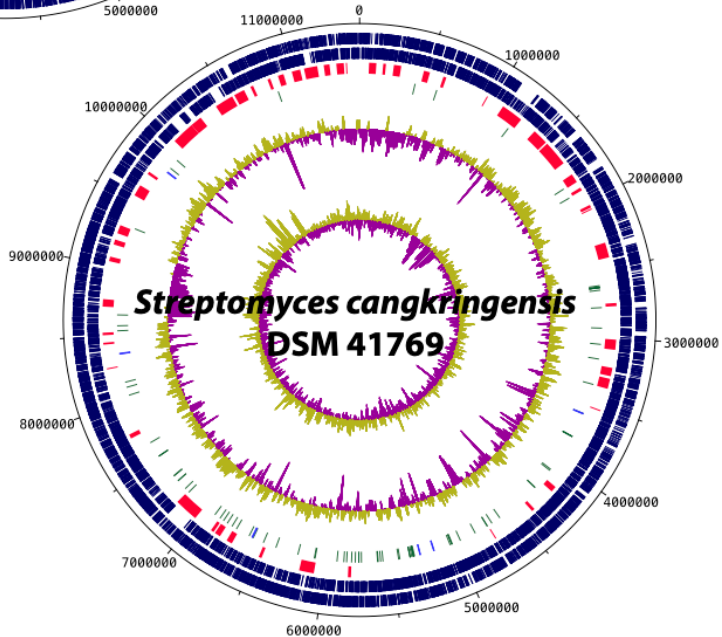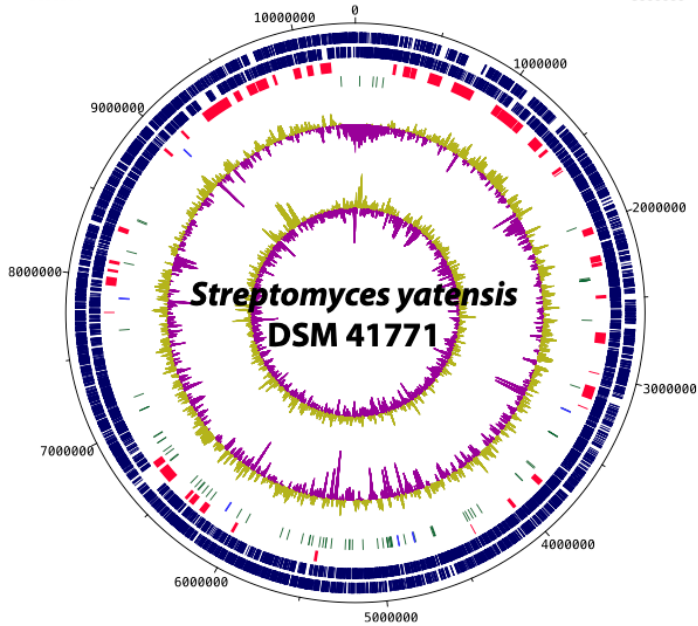

Supplement: FIG S1 [file msystems.00489-21-sf001.pdf]

a. Subgroup F3

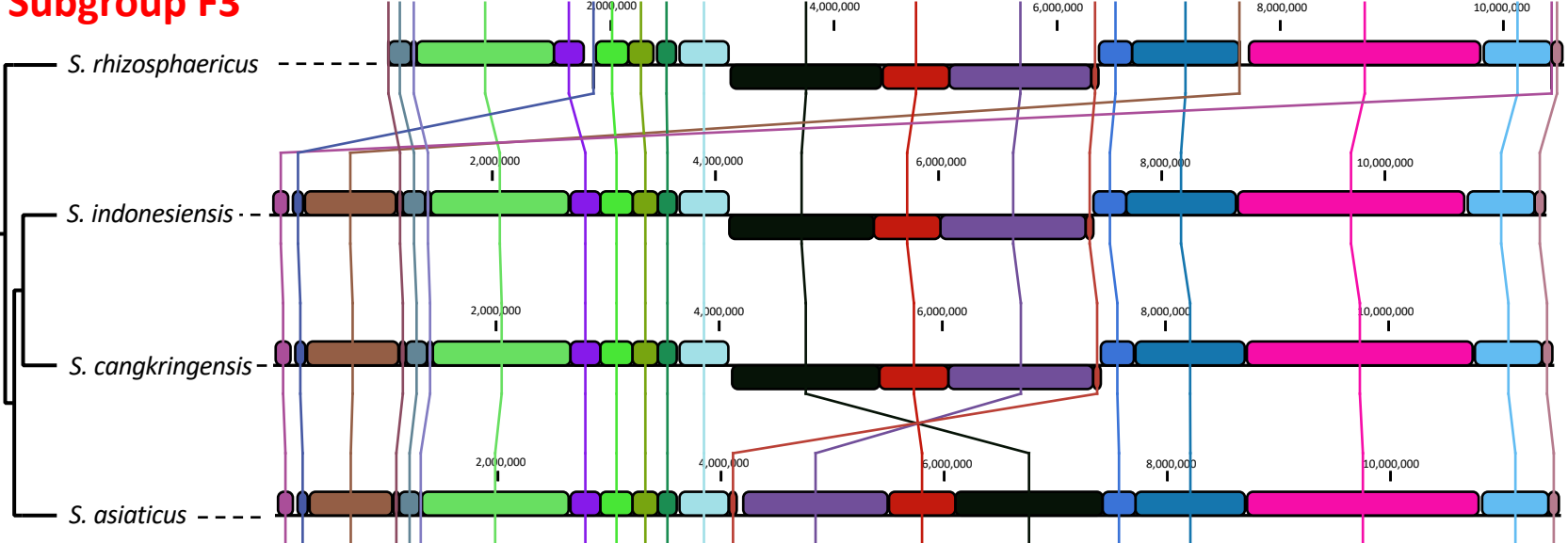

b. Subgroup F1

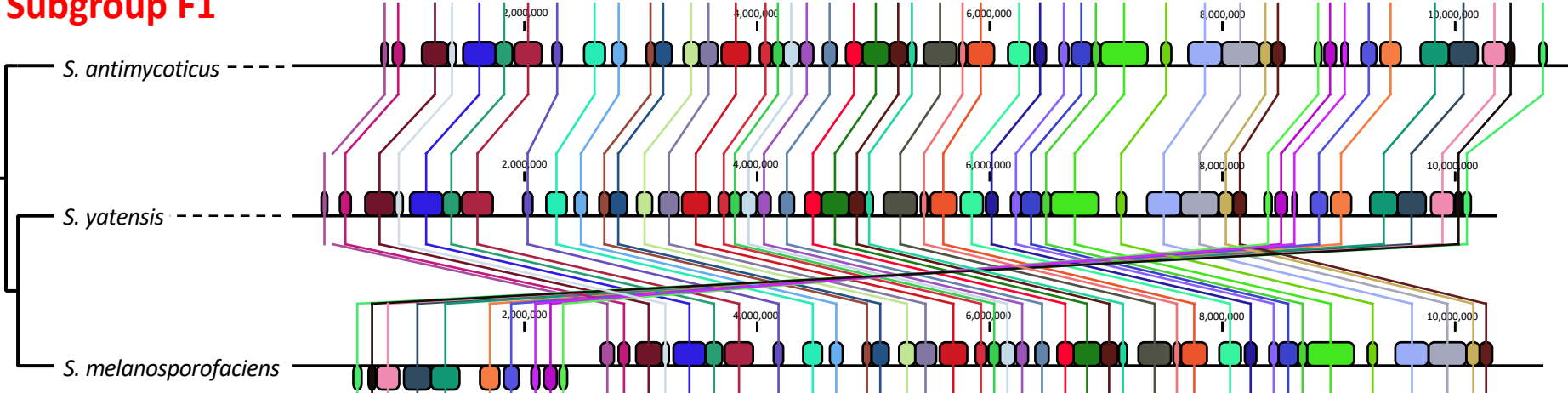

Supplement: FIG S2 [file msystems.00489-21-sf002.pdf]

common GCFs

rare GCFs

unique GCFs

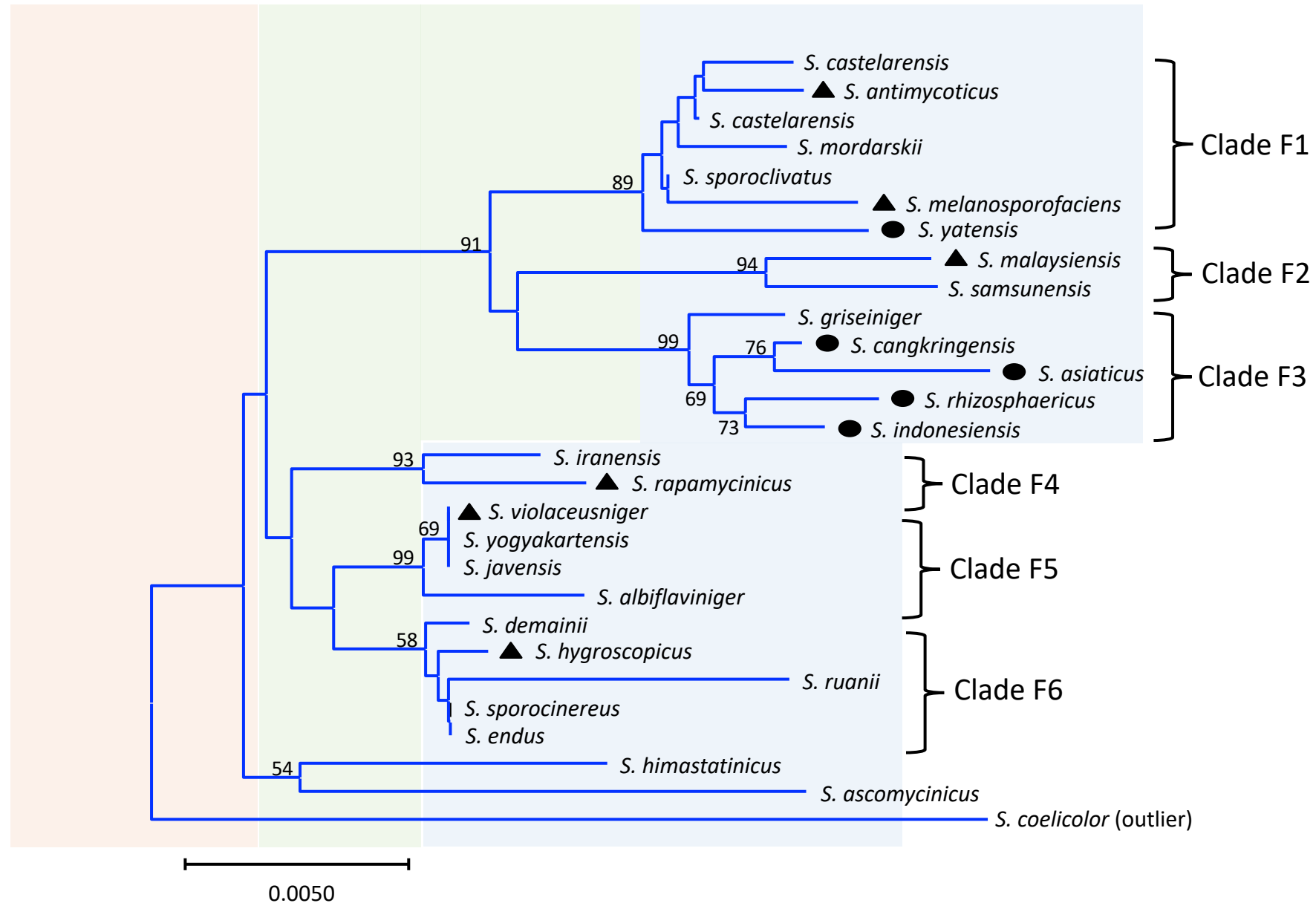

Supplement: FIG S3 [file msystems.00489-21-sf003.pdf]

a. **GCF-10: T1PKS**

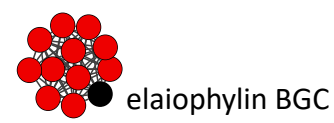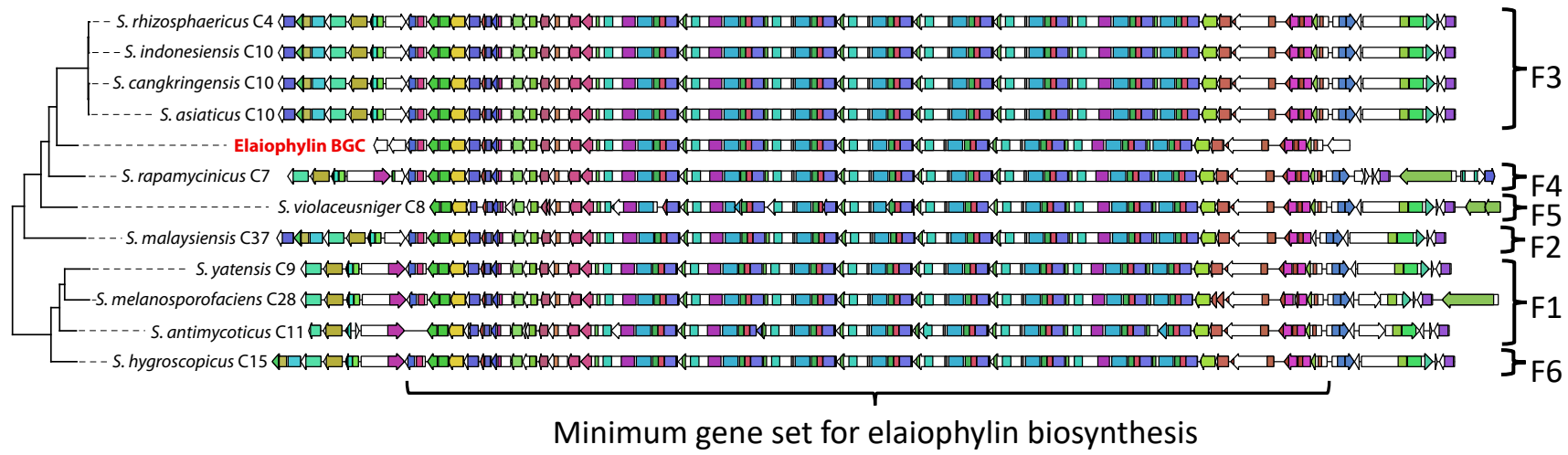

b.

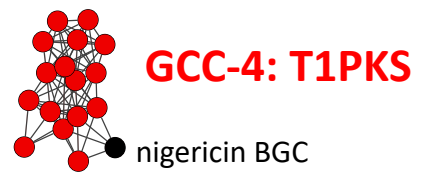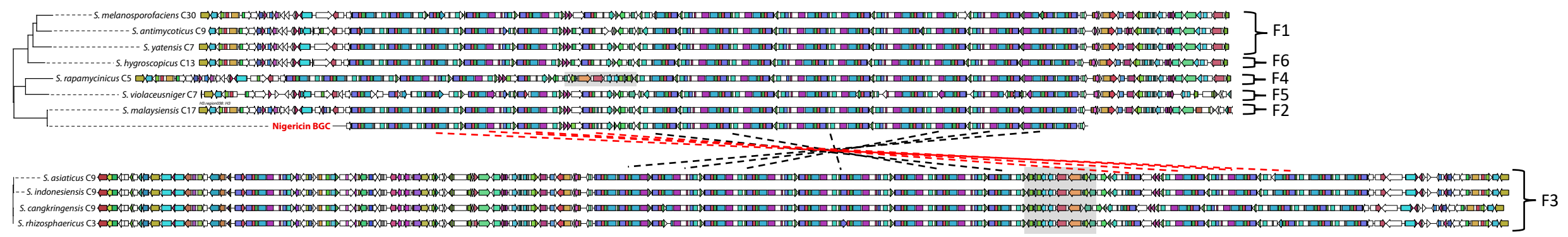

Supplement: FIG S4 [file msystems.00489-21-sf004.pdf]

a. **GCC-5: T1PKS**

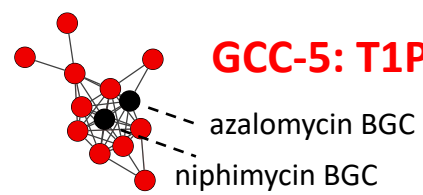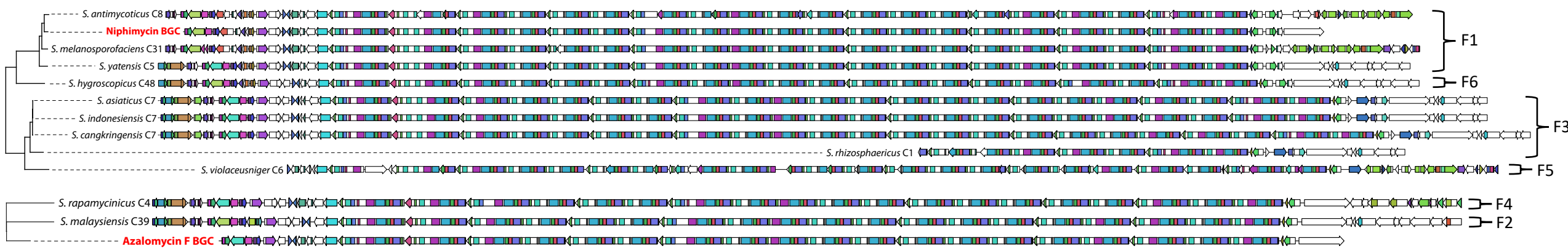

b. **GCF-6: T1PKS**

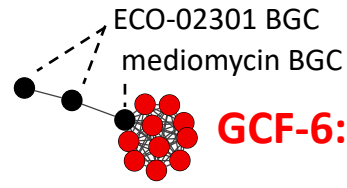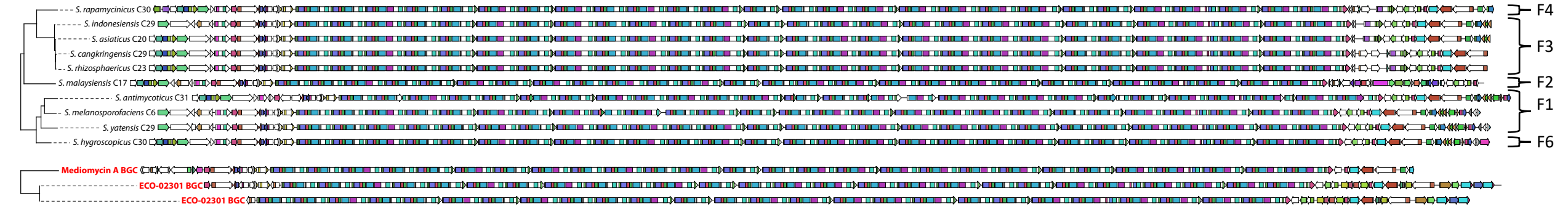

Supplement: FIG S5 [file msystems.00489-21-sf005.pdf]
